# Automatic Classification of the Sub-Techniques (Gears) Used in Cross-Country Ski Skating Employing a Mobile Phone

**DOI:** 10.3390/s141120589

**Published:** 2014-10-31

**Authors:** Thomas Stöggl, Anders Holst, Arndt Jonasson, Erik Andersson, Tobias Wunsch, Christer Norström, Hans-Christer Holmberg

**Affiliations:** 1 Department of Sport Science and Kinesiology, University of Salzburg, Hallein/Rif 5400, Austria; E-Mail: tobias.wunsch@sbg.ac.at; 2 Swedish Winter Sports Research Centre, Department of Health Sciences, Mid Sweden University, Östersund 83140, Sweden; E-Mails: erik.andersson@miun.se (E.A.); Hans-Christer.Holmberg@miun.se (H.-C.H.); 3 Swedish Institute of Computer Science, Kista 16440, Sweden; E-Mails: aho@sics.se (A.H.); arndt@sics.se (A.J.); cn@sics.se (C.N.); 4 School of Computer Science and Communication, Royal Institute of Technology, Stockholm 10044, Sweden; 5 Swedish Olympic Committee, Stockholm 11433, Sweden

**Keywords:** algorithm, collective classification, gaussian filter, individual classification, markov chain, machine learning, smartphone

## Abstract

The purpose of the current study was to develop and validate an automatic algorithm for classification of cross-country (XC) ski-skating gears (G) using Smartphone accelerometer data. Eleven XC skiers (seven men, four women) with regional-to-international levels of performance carried out roller skiing trials on a treadmill using fixed gears (G2left, G2right, G3, G4left, G4right) and a 950-m trial using different speeds and inclines, applying gears and sides as they normally would. Gear classification by the Smartphone (on the chest) and based on video recordings were compared. Formachine-learning, a collective database was compared to individual data. The Smartphone application identified the trials with fixed gears correctly in all cases. In the 950-m trial, participants executed 140 ± 22 cycles as assessed by video analysis, with the automatic Smartphone application giving a similar value. Based on collective data, gears were identified correctly 86.0% ± 8.9% of the time, a value that rose to 90.3% ± 4.1% (*P* < 0.01) with machine learning from individual data. Classification was most often incorrect during transition between gears, especially to or from G3. Identification was most often correct for skiers who made relatively few transitions between gears. The accuracy of the automatic procedure for identifying G2left, G2right, G3, G4left and G4right was 96%, 90%, 81%, 88% and 94%, respectively. The algorithm identified gears correctly 100% of the time when a single gear was used and 90% of the time when different gears were employed during a variable protocol. This algorithm could be improved with respect to identification of transitions between gears or the side employed within a given gear.

## 1. Introduction

New technologies, such as mobile Internet and Smartphones, with sensors, social media and networking, offer novel opportunities for developing intelligent tools for use in sports science and training. The introduction of the iPhone in 2007 opened a new era of user-IT interface, including thousands of mobile phone applications (apps), that has significantly altered the relationship of ordinary people to advanced technology, making it an integrated part of their daily lives.

These advances led to the first generation of apps designed for sports and fitness, including apps that monitor routes, distances and styles of running, technique in various sporting activities, and fitness or training records. Both easy to use and readily accessible, these apps utilize the internal sensors and actuators in Smartphones, including GPS, accelerometers, cameras and sensors of sound and vibration, providing immediate and high-impact display of the results. These same internal sensors might allow recording, analysis and presentation of biomechanical and physiological data that are routinely collected employing much more complex methodology.

Accordingly, these developments might move the testing of athletes from the artificial environment of sports laboratories out into the field and at a significantly lower cost as well. In addition, the data collected can be transmitted in real-time to cloud-based services for discerning patterns, providing immediate rather than the delayed feedback associated with laboratory monitoring.

Movement patterns have been classified in various contexts before, for example by an accelerometer that monitors whether an elderly person is standing, sitting, or lying down in order to sound an alarm if he/she falls [1]. In addition, there have been attempts to classify swimming styles using microcomputer-based recording of acceleration [2] or the dynamics of ski jumping during takeoff and stable flight on the basis of data from inertial measurement units (IMUs) placed on the ski jumper and equipment [3,4]. Moreover, researchers have employed the internal sensors of a Smartphone to study the movements of hockey and soccer players [5], characterize activities such as walking or running [6], and classify basic physical activities such as sitting, lying, standing, walking, jogging, walking up- and downstairs, *etc.*, and the transitions between these [7,8].

In connection to endurance sports, the speed, duration and intensity (heart rate, blood level of lactate) of a training session are often of interest. However, cross-country (XC) skiing, for example, involves several different techniques, divided mainly into classical and skating techniques, each of which includes different patterns of motion, known as gears. In the case of the skating technique, skiers use four main sub-techniques referred to as gears 2–5 (G2–5) [9]. The lower gears are utilized on uphill terrain and the higher gears on easier terrain at higher velocities or under conditions of lower ski-snow friction.

Furthermore, depending on the terrain and/or personal preference, skiers can chose to rely primarily on either the right or left pole while skating, a situation often referred to as the strong or dominant and weak or non-dominant sides [10–13]. In connection with the G2 and G4 sub-techniques athletes and coaches often debate whether both sides should be developed almost equally in connection with training sessions. Accordingly, enhanced understanding of which gears are used on different parts of a track and the characteristics (e.g., cycle rate) associated with each sub-technique should allow more effective training strategies.

Accelerometers are rarely utilized to collect data about XC skiing [5,14–16] and with the exception of the pre-study by Holst and Jonasson [17], to the best of our knowledge, integrated Smartphone sensors were not yet employed for automatic XC skiing gear identification. It has been demonstrated that the various skiing gears can be distinguished from one another by visual inspection of accelerometer data collected from a single location on the body [18]. One difficulty in this connection arises from the quite subtle differences between the skiing gears. Walking and running involve significant differences in amplitude, as well as frequency. However, one and the same skiing gear can be employed either rapidly or slowly, on different terrain, and with varying intensity, so amplitude and frequency are not distinct features. Thus, a model that distinguishes between the different gears should be essentially independent of these factors, as well as, for maximal utility, applicable to different skiers, both professional and amateur, and with the different and varying sampling frequencies of various mobile phones.

The specific aims of the current investigation were to develop and validate an algorithm that can employ accelerometer data collected by a Smartphone to classify each gear of XC skating skiing, independent of the level of performance of the skier.

## 2. Methods

### 2.1. Participants

Eleven XC skiers (seven men and four women) who competed at regional-to-international levels volunteered to participate. All were fully informed of the nature of the investigation prior to providing their written consent to participate and the study was pre-approved by the Regional Ethical Review Board.

### 2.2. Overall Design

First, each participant skied at a fixed speed and incline using each gear (G2, G3, G4), with the right or left side being dominant during G2 and G4 (G2L, G2R, G4L, G4R), following a standardized protocol. Thereafter, each participant rollerskied 950 m on a treadmill at different speeds and inclines using his/her preferred gear and switching between sides when using G2 and G4—once for familiarization and a second time for collection of data. During all trials acceleration was monitored by a Smartphone attached to the chest of the participant. Two approaches to gear classification were utilized: (1) individual classification of gears on the basis of the data from the first trial (the fixed gears protocol by each participant) and (2) classification based on collective data obtained in advance during various training sessions on snow and roller skis by different skiers. For validation, a video camera (Sony, 50 Hz, Tokyo, Japan) situated at the rear end of the treadmill, with a view of the complete frontal plane of the skier and the entire range of pole movement, was employed to identify gear selection and cycle characteristics (the exact time at which each cycle was started and the total number of cycles with the different gears and for the entire trial). Classification of gears on the basis of the Smartphone data was performed without knowledge about the gears actually being employed.

### 2.3. Detailed Experimental Protocol

After warming up by skiing for 5 min at ∼70%–85% of VO_2max_, each skier performed two sets of six trials each under conditions employed during low-intensity training on the treadmill: (1) with G3 at 15 km/h on essentially flat terrain (incline 1°); (2) G3; (3) G2L and (4) G2R, all at 12 km/h on a moderate incline (5°); and (5) G4L and, finally, (6) G4R, both at 15 km/h on essentially flat terrain (1°). For each trial approximately 50 cycles were monitored. The data from the first trial were utilized for machine learning designed to ensure reliable application of the algorithm to each individual skier. On the basis of the second trial the accuracy of the algorithm for classifying the gear being applied by a single skier under standardized conditions was evaluated.

After a 3-min break, each participant then performed the variable treadmill protocol twice, with a 5-min rest between trials. The basic protocol consisted of (1) a 350-m flat section (1°) (200 m at 15 km/h and 150 m at 20 km/h); (2) 225 m of moderate incline (150 m at 3°, 13 km/h, and 75 m at 5°, 11 km/h); (3) another 200-m flat section (1°, 20 km/h); and (4) the final 75-m climb (9 km/h at 7°). The treadmill speed was maintained constant by a laser system installed at the rear end, unless the skier moved to the front or rear of the belt, in which case the speed was automatically increased or decreased, thereby allowing each participant to match the intensity to their individual moderate training speed.

### 2.4. Instrumentation

Each participant used his/her own poles and boots, but the same pair of Pro-Ski C2 rollerskis (Sterners, Nyhammar, Sweden). The treadmill belt (Rodby, Södertalje, Sweden) was large enough (3.3 × 2.5 m) to allow roller skiing with the skating technique and all participants were accustomed to roller skiing at various speeds and inclines on this treadmill as part of both their training and testing. During all testing the participants were secured with a safety harness connected to an emergency brake suspended above the treadmill.

### 2.5. The Smartphone and Software

Using a Sony Ericsson Xperia ST17i attached to the front of the chest (centrally above the sternum) with a specially designed belt and oriented so that the *x* axis was lateral, the *y* axis vertical, and the *z*-axis horizontal, acceleration was sampled at 80 Hz. Accelerometer data were stored on the Smartphone for subsequent processing.

### 2.6. The Algorithm

The general approach was, first, to employ the continuous three-dimensional readings from the Smartphone to identify each cycle of movement and transform it into a normalized representation of fixed length, and thereafter to utilize machine learning to classify these cycles.

### 2.7. Data Preprocessing

Since the accelerometer data were rather noisy, a simple averaging low-pass filter consisting of Gaussian convolution was applied as a first step. The advantages of a Gaussian filter in the present context are the absence of ringing effects; no undesirable time shifts at different frequencies; simple and rapid computation; and, most importantly, reliability in the face of the varying and sometimes irregular rates of sampling by the Smartphone. For identification of single skating cycles, a Gaussian convolution with a standard deviation of 370 ms was utilized to divide the very-low-frequency signal into cycles. Subsequently, the raw signal for each individual cycle was filtered with a Gaussian for somewhat higher frequencies (90-ms standard deviation) to retain significant features while removing the noise. At the same time, each cycle was resampled in 100 steps, to obtain a fixed-length vector of 300 dimensions, *i.e.*, 100 accelerations each along the *x*-, *y*- and *z*-axes. This vector represents a trajectory in the acceleration space during one cycle.

### 2.8. The Statistical Model

A detailed description of the machine-learning model involved has been provided by Holst and Jonasson [17]. In brief, to represent the cycles for classification of the individual skating gears, a statistical machine-learning model consisting of a Markov chain of multivariate Gaussian distributions was applied, because of its advantages in connection with this specific task. For example, a nearest-neighbor classifier does not take significant differences or correlations between the elements in the vector into consideration; whereas a Gaussian classifier with a full covariance matrix can model correlations and significance correctly, but has too many degrees of freedom to learn from the limited amount of data available for each gear.

On the other hand, a Markov model has fewer parameters while still being sufficiently complex to accurately represent the trajectory of movement. Furthermore, a Markov model of Gaussian distributions avoids the problem of making the input discrete, which, depending on the number of steps involved, would either result in the loss of important details and/or require a prohibitively large number of parameters. Thus, the model we selected has just a large enough number of parameters to represent the relevant aspects of the task, providing potentially high generalizability, as well as robustness with respect to the limited amount of training data collected. Both the preprocessing and the classification algorithm were run on a laptop in C++.

### 2.9. Training Data

The training data used for collective classification were collected during seven different training sessions on both snow and roller skis, performed prior to the start of the current study by one–13 athletes. For the individual classification, data from the first trial by each participant using the fixed gears protocol was utilized.

### 2.10. Statistical Analyses

The classified gears (based on data from the accelerometers) and actual gears (based on video analysis) are presented in both absolute and relative terms (% of total use of the gear in question) in the form of a confusion matrix and a paired-sample t-test applied to compare the accuracy of these two approaches. Pearson's Product moment correlation coefficient was calculated between the gears classified correctly and the number of transitions during the trial involving individualized use of gears. For all analyses, the level of statistical significance was set at α = 0.05. All statistical analyses were carried out using the SPSS 22.0 Software (SPSS Inc., Chicago, IL, USA) and Office Excel 2010 (Microsoft Corporation, Redmond, WA, USA).

## 3. Results

**Figure 1.**
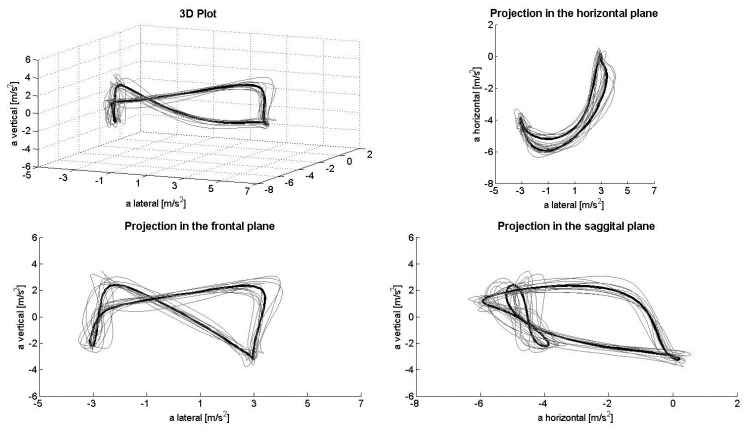
Acceleration during G2L in the total space (**upper left**) and frontal (**lower left**), horizontal (**upper right**) and sagittal planes (**lower right**). These data have been filtered with a Gaussian for higher frequencies (90-ms standard deviation). Due to movement of the trunk (where the Smartphone was attached) within a cycle, the acceleration signals do not always represent the true components (especially in the vertical and horizontal directions).

**Figure 2.**
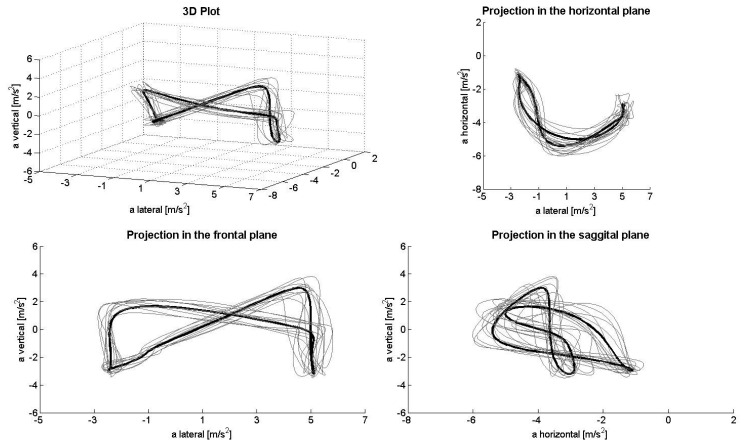
Acceleration data during G2R in the total space (**upper left**) and frontal (**lower left**), horizontal (**upper right**) and sagittal planes (**lower right**).

**Figure 3.**
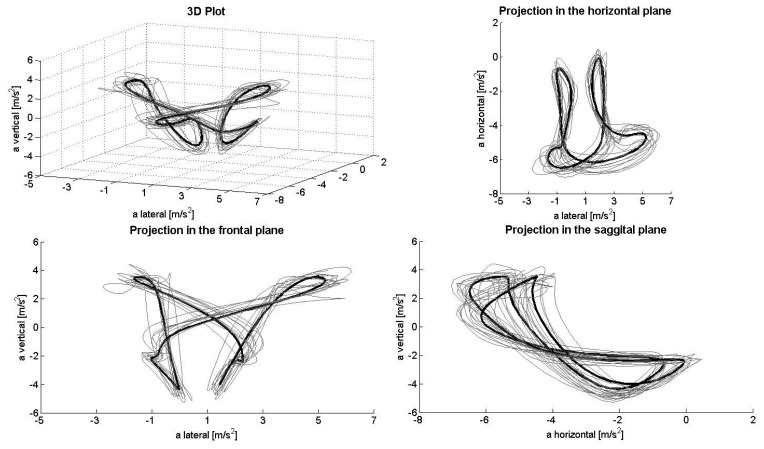
Acceleration data during G3 in the total space (**upper left**) and frontal (**lower left**), horizontal (**upper right**) and sagittal planes (**lower right**).

**Figure 4.**
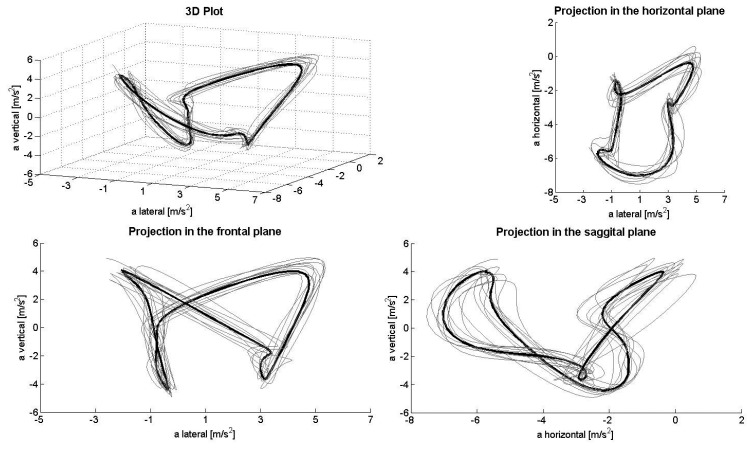
Acceleration data during G4L in the total space (**upper left**) and frontal (**lower left**), horizontal (**upper right**) and sagittal planes (**lower right**).

**Figure 5.**
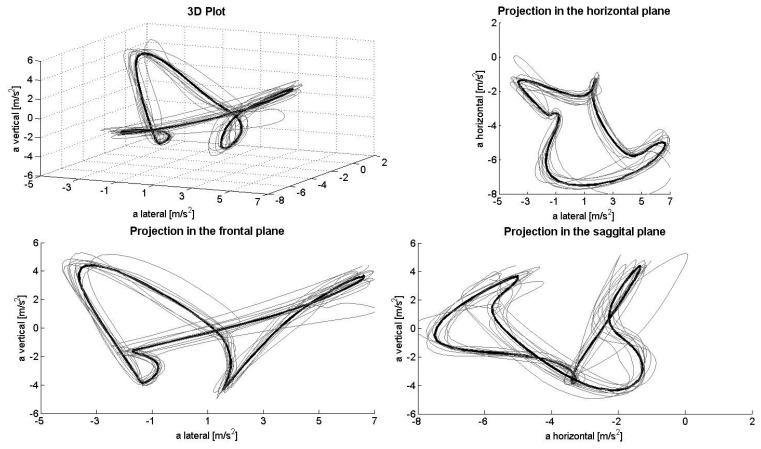
Acceleration data during G4R in the total space (**upper left**) and frontal (**lower left**), horizontal (**upper right**) and sagittal planes (**lower right**).

Figures 1, 2, 3, 4 and 5 depict the filtered acceleration data in the sagittal, frontal and horizontal planes for each gear, along with three-dimensional plots. Distinct symmetric and asymmetric patterns (such as the “*half moon*” in the horizontal plane during G2 or almost symmetric “*butterflies*” in the horizontal and frontal planes during G3) are discernible. Therefore, the input data appear to contain the information required for a machine-learning algorithm to solve the task.

**Figure 6.**
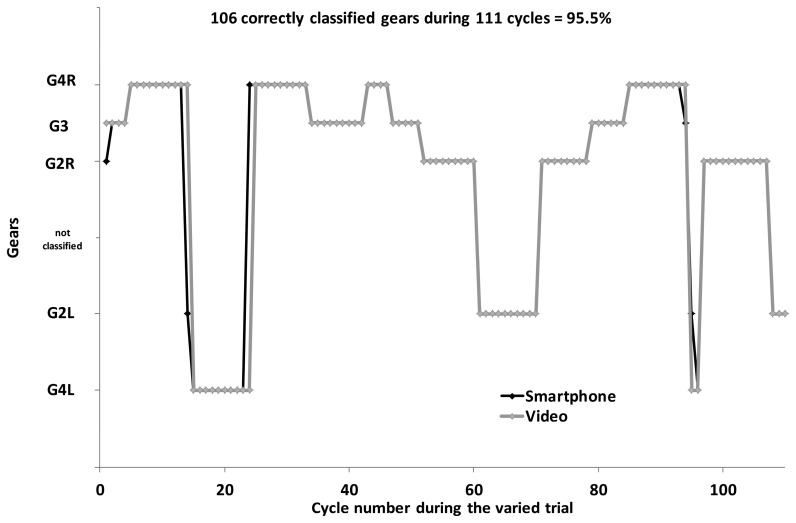
Comparison of automated gear classification using acceleration data from the Smartphone (black line and dots) with the video analysis (grey line and dots) for one of the best skiers.

For all trials using the fixed gear, incline and speed, the Smartphone application identified the gears correctly 100% of the time for all of the participants, using either the collective or individual data in the algorithm. During the variable 4-min XC skiing trial, the participants executed a total of 140 ± 22 cycles (range: 111–173), 30 ± 13 with G2L, 27 ± 13 with G2R, 30 ± 13 with G3, 26 ± 9 with G4L, and 26 ± 13 with G4R, as well as 23 ± 12 transitions between gears, as revealed by the video analysis used as a gold standard. The corresponding values obtained with the automatic Smartphone application were similar (e.g., Figure 6).

With the collective data set gear identification was correct 86.0% ± 8.9% of the time (range: 61–94), a value that rose to 90.3% ± 4.1% (82%–97%) (*P* < 0.01) when the individual data were utilized. For nine of the 11 participants correct detection was >90%, while for the other two skiers only 82% and 85%. For these latter two participants, who switched between gears and/or sides within a single gear unusually often, gear identification was most often incorrect during transitions between gears, especially when switching to or from G3. In general, the degree of correct detection was negatively correlated to the number of transitions between gears (r = −0.66, *P* < 0.05). The numbers of incorrect identifications immediately after commencement of measurement and during the transition phase between individual gears were 2 ± 3 (14% ± 17% of the total number of incorrect identifications) and 8 ± 6 (61% ± 34% of the total number), respectively, thus representing together 75% ± 37% of the gears classified incorrectly. The confusion matrix for the algorithm based on individual data demonstrates the degrees of correct and incorrect identification in absolute and relative terms (Table 1). The accuracies of the automatic procedure for identifying G2L, G2R, G3, G4L and G4R were 96%, 90%, 81%, 88% and 94%, respectively.

## 4. Discussion

Classification of XC ski-skating gears via a Smartphone-based algorithm was correct more than 90% of the time. Clearly, a statistical machine-learning procedure based on a Markov chain of multivariate Gaussian distributions can use accelerometer readings (e.g., from a mobile phone attached to a belt around the chest) alone to classify periodic patterns of movement during XC skiing. It is not surprising that such a Markov model, which is designed specifically for sequential patterns, is in many ways better suited to classify patterns of movement than traditional monitoring of amplitude or frequency. This method is relatively unaffected by noise and can deal effectively with differences in rates of data collection by the hardware, as well as differences between individual skiers, making it highly suitable for practical use.

Approximately 75% of the incorrect classifications of gears occurred at the beginning of the test trials, especially during transitions between different gears. In addition, identification of the gears was most accurate for skiers who made fewer transitions between gears. Furthermore, during all the trials involving a constant predefined gear, with no transitions, identification was 100% accurate, which might well reflect a more consistent pattern of acceleration when utilizing any single gear.

These findings probably reflect differences in the patterns of acceleration when switching between gears (G2–G4) or between the preferred and non-preferred sides within a single gear (e.g., G2 and G4). For instance, change of the preferred side when employing the G4 technique involves a variant of the G3 technique, with a more pronounced movement of the arm during approximately the final 25% of the cycle. In addition, the switch between sides within G2 represents yet another variant of G3 with, due to the asymmetric motion [13], slightly altered positions of the arm and trunk. Indeed, poorest identification was associated with switching to or from G3, so here is the greatest potential for improvement through the use of machine learning. Consequently, not only the gear being utilized, but also transitions between gears and even between dominants side (e.g., G2L to G2R, G2L to G3, G2R to G3, *etc.*) should be employed for classification.

Identification by the algorithm based on the collective data alone was somewhat less accurate than that provided by machine learning using individual data (86% *versus* 90%). This is in line with findings involving other sensors; for example, foot pots for determination of running speed must be calibrated individually for optimal performance. However, for practical use “calibration” of the algorithm for individual gear classification involves a standardized procedure comparable to the one presented here, which must be carried out at least once and often several times (e.g., when the individual technique changes).

Two earlier investigations have demonstrated the usefulness of inertial sensors for *post hoc* identification of sub-techniques employed in XC skiing. One of these utilized a unit consisting of an accelerometer, gyroscope, GPS and magnetometer sensor to identify sub-techniques of the classical and skating styles [18] and the other five triaxial accelerometers to characterize the differences between G2 and G3 [14]. With the exception of the pre-study by Holst and Jonasson [17], where both a Smartphone and Zephyr Bioharness were utilized, the present study is, to the best of our knowledge, the first to employ the integrated accelerometer sensors of a Smartphone in combination with an automatic algorithm to identify gears of XC skiing. The advantages of this approach include (1) the possibility to record, process, visualize and document the data collected with a single apparatus; (2) the ability to also use various other sensors; (3) utilization of cloud-based features through direct connection to the Internet; and (4) the fact that a Smartphone is usually carried somewhere on the body of the user (in a pocket, on a belt, in a special constructed harness, *etc.*) both during daily living, but also during sporting activities.

### Perspectives and Limitations

A simple procedure for identifying XC skiing gears, such as the one described here, is an important first step towards analysis of the use of different gears on the various sections of an entire race track. Moreover, it should be possible to refine this approach to provide various indices of performance, including consistency of movement, acceleration/velocity in the direction of skiing and any left-right asymmetries in movements. One example of slight asymmetries in connection with G3, which is, at least in theory, a symmetrical technique, can be seen in Figure 3. Furthermore, different skiers can be compared in order to identify optimal movements within each gear and individual skiers can receive immediate feedback in connection with their training. Finally, the same approach could be applied to skiing with classical techniques and perhaps to any sport or activity involving periodic movements. However, the algorithm applied here was found to be valid under optimal standardized conditions, *i.e.*, during treadmill rollerskiing, and it remains to be determined whether this is also the case for skiing on snow. In particular, skiing through curves and/or around corners might make classification of patterns of acceleration more difficult.

The procedure applied here detected >90% of the gears being utilized both correctly and automatically. At the same time, analysis of acceleration data along the three axes, especially in the horizontal direction, appeared to be somewhat faulty. In theory, when skiing on a treadmill, the athlete remains roughly in the same location, so that the second integral of the acceleration (*i.e.*, the distance) covered during a cycle should equal zero. However, as can be seen, for example, in the projection plots for the sagittal plane, the horizontal component demonstrates only negative acceleration, while the lateral component displays a slight positive shift. Thus, a double integral of these data would indicate significant accumulated displacement in the forward/backward and lateral directions. This error might result from a combination of (1) the fixed alignment of the Smartphone on the body; (2) the effect of gravity on the coordinate system of the sensor; and (3) errors in the alignment of the local and global coordinate systems due to extension/flexion and rotation of the trunk during a cycle (*i.e.*, when the trunk is bent, the vertical component of gravity will be confused with horizontal acceleration). To avoid this problem, the coordinate systems can be stabilized using other sensors, such as a gyroscope or magnetometer. However, as the present study shows, this is not necessary for purposes of classification only, at least not during rollerskiing on a treadmill.

## 5. Conclusions

An algorithm involving Gaussian filters and a Markov-chain machine learning procedure correctly identified the gear being used for XC ski-skating 100% of the time when a single gear was utilized and 90% of the time when the gear was being varied. Possible future improvements include more accurate characterization of transitions between gears and alteration of the preferred side within a gear, perhaps based on monitoring individual patterns of acceleration with each gear. Furthermore, the algorithm remains to be validated in connection with XC skiing on snow. In addition, the next generation of intelligent training applications might provide information about symmetry/asymmetry, as well as changes in and the economy of movement, thereby supplying direct feedback, diagnostics and documentation of training to both elite and recreational athletes and coaches. In addition to providing valuable information to the skiers themselves, certain key indices of performance can be made available to spectators (by television and/or the internet) in novel ways. In addition to seeing the facial expressions and poses of the athletes, heart rate, cycle frequencies, the pattern of gear use and other technical matters can be analyzed by commentators, thereby making the spectator experience even more dramatic and rewarding.

## Figures and Tables

**Table 1.** The confusion matrix (n, %) for the gears employed during XC ski skating as identified by video analysis *versus* the smartphone application (mean value, *n* = 11).

**Video Analysis**	**Smartphone Analysis**				

	**G2L**	**G2R**	**G3**	**G4L**	**G4R**
G2L	28 (96%)	3 (7%)	1 (3%)	2 (3%)	1 (0%)
G2R	1 (2%)	23 (90%)	2 (4%)	1 (2%)	0 (0%)
G3	1 (1%)	1 (1%)	25 (81%)	3 (6%)	2 (4%)
G4L	4 (1%)	0 (0%)	2 (4%)	23 (88%)	2 (2%)
G4R	1 (0%)	3 (2%)	3 (8%)	2 (2%)	25 (94%)

G, gear; L, left; R, right.
